# Nonexudative Macular Neovascularization in Age-Related Macular Degeneration

**DOI:** 10.1001/jamaophthalmol.2026.0459

**Published:** 2026-04-09

**Authors:** Sridevi Thottarath, Sarega Gurudas, Syed Kubravi, Dimitrios Kazantzis, Ayse Merve Keskin, Sobha Sivaprasad

**Affiliations:** 1 Moorfields Clinical Research Facility, NIHR Biomedical Research Centre, Moorfields Eye Hospital NHS Foundation Trust, London, United Kingdom; 2 University College, London, United Kingdom; 3 University Hospital Bristol NHS Foundation Trust, Bristol, United Kingdom

## Abstract

**IMPORTANCE:**

Nonexudative macular neovascularization (neMNV) is a risk factor for exudation in fellow eyes of patients with unilateral exudative age-related macular degeneration (AMD). Accurate estimation of prevalence of neMNV is required for sample size calculations for clinical trials evaluating novel interventions to delay exudation from neMNV. Certain dimensions of double-layer sign (DLS) on optical coherence tomography (OCT) are often regarded as a surrogate for neMNV.

**OBJECTIVE:**

To assess the prevalence of fellow-eye neMNV on OCT angiography (OCT-A) in eyes with AMD with DLS on OCT among patients with unilateral new-exudative AMD in the first eye.

**Design, Setting, and Participants:**

This prospective observational cohort multicenter study took place in the United Kingdom from January 2021 through June 2025. The study included 862 participants with OCT and OCT-A within 3 months of initiation of anti–vascular endothelial growth factor (VEGF) therapy, of whom 550 (63.8%) had both OCT and OCT-A performed within 30 days of the date of the first anti-VEGF injection to the fellow eye. These data were analyzed from July 2025 through October 2025.

**MAIN OUTCOMES AND MEASURES:**

Prevalence of DLS, neMNV, and assessment of univariable and adjusted associations with neMNV and DLS were assessed using logistic regression models.

**RESULTS:**

Among 550 eyes (mean [SD] participant age, 78.0 [7.6] years; 315 female [57.3%] and 235 male [42.7%]), 112 (20.4%; 95% CI [Wilson score], 17.1%-24.0%) had DLS and 47 eyes (8.5%; 95% CI [Wilson score], 6.4%-11.3%) had neMNV at baseline, including 42 (89.36%) within DLS, 3 (6.4%) in fibrovascular irregular shallow–pigment epithelial detachment, and 2 (4.3%) below drusen. Prevalence of neMNV was higher among eyes with thick DLS (n = 36 [48%]) compared with those with thin DLS (16.2%; n = 6; difference = −31.8%; 95% CI [Wilson score], −46.9% to −11.7%; *P* = .002).

**CONCLUSIONS AND RELEVANCE:**

While 1 in 5 fellow eyes had DLS on OCT among patients with unilateral new-onset exudative AMD in the first eye, the prevalence of neMNV on OCT-A was only 40% among these eyes with DLS. These results suggest DLS on OCT in fellow eyes of patients with new-onset exudative AMD in the first eye is not a good surrogate for neMNV

**Trial Registration:**

ISRCTN registry Identifier: ISRTCTN13798759

## Key Points

**Question:**

What is the prevalence of fellow-eye nonexudative macular neovascularization (neMNV) on optical coherence tomography angiography (OCT-A) in age-related macular degeneration (AMD) eyes with double-layer signs (DLS) on OCT among patients with unilateral new exudative AMD in the first eye?

**Findings:**

In this prospective cohort study, 1 in 5 fellow eyes had DLS. However, only 40% with DLS had neMNV on OCT-A.

**Meaning:**

These results suggest DLS on OCT in fellow eyes of patients with new-onset exudative AMD in the first eye should not be used as a surrogate for neMNV in that fellow eye.

## Teaser

This study assesses the prevalence of fellow-eye nonexudative macular neovascularization on optical coherence tomography-angiography in eyes with age-related macular degeneration with double-layer signs on optical coherence tomography among patients with unilateral new exudative in the first eye.

## Introduction

Exudation from active neovascular age-related macular degeneration (nAMD) is a leading cause of visual impairment in individuals aged 50 years and older in developed countries.^1^ Most patients present with unilateral exudative nAMD. The second eyes are at risk of transitioning to exudative nAMD but the reported conversion rates vary between 12% to 22% at 2 years and 22% to 38.7% over 5 years.^2,3,4,5^

The presence of subclinical nonexudative macular neovascularization (neMNV) on optical coherence tomography angiography (OCT-A) is a strong risk factor for exudation.^6,7,8^ These asymptomatic neMNVs may occur at any stage of nonneovascular AMD, as evidenced in histopathology examination of postmortem eyes.^9^ Previous reports have shown wide variation in estimated prevalence of neMNV in fellow eyes of patients with unilateral exudative AMD ranging from 6.25% to 27.00%, mostly explained by the time point at which a participant is included in the study from the date of diagnosis of exudative nAMD in the fellow eye.^10,11^ In addition, neMNV ranks as a variable predictor in recent risk models for new-onset active nAMD, with incidence of exudation ranging from 20% to 80% within 2 years of follow-up.^11,12,13^ There is also inconsistent evidence that neMNV may slow down the growth of adjacent geographic atrophy.^14,15,16^

However, most studies evaluated the prevalence of double-layer sign (DLS) on optical coherence tomography (OCT) as a surrogate for neMNV because OCT of the fellow eye is done regularly in routine clinical practice when treating the first eye.^17,18,19^ The DLS is defined as a shallow separation of the retinal pigment epithelium (RPE) from the Bruch membrane of less than 100 μm in height.^18,19,20,21^ Heterogeneous reflectivity of the content of DLS is a presumed sign of neMNV. Previous reports have suggested that DLS of more than 1000 μm length, otherwise termed *shallow irregular RPE elevation*, are more likely to have neMNV.^18^ The reflectivity of the contents of the DLS is not always gradable if there is only a minimal separation, often categorized as thin DLS.^22,23^ A shallow RPE detachment with a height just over 100 μm can also harbor neMNV. These may be better termed as *flat-irregular RPE detachments* (FIPED), as seen in central serous chorioretinopathy.^24^ Irregular elevations of RPE may not all be due to neMNV as they can be occupied by basal laminar deposits (BLD), small confluent drusen, cuticular drusen, or even unknown nonreflective material.^14,23^ Therefore, it is unclear whether DLS could be used as a surrogate for neMNV, especially in clinical trials evaluating interventions that may prevent exudation from neMNV.

The EYENEON study evaluated the prevalence of neMNV and DLS in the fellow eyes of patients with unilateral new-onset exudative nAMD. As a subgroup analysis, the dimensions of DLS that is most likely to harbor neMNV and the associations of DLS and neMNV were also determined.

## Methods

This multicenter cohort study was conducted across 25 National Health Service sites adhering to the tenets of the Declaration of Helsinki and approved by the UK London-City and East National Research Ethics Committee (20/PR/0897). Written informed consent was obtained from all participants. Participants did not receive a stipend or other incentive to participate. This report follows the Strengthening the Reporting of Observational Studies in Epidemiology (STROBE) reporting guidelines. The EYENEON protocol has been published previously.^25^

### Participants

Patients aged 50 years and older and younger than 100 years with a diagnosis of unilateral treatment-naive exudative neovascular AMD with available OCT and OCT-A within plus or minus 3 months of initiation of anti-vascular endothelial growth factor (VEGF) therapy were included. The fellow (second) eye with any stage of nonneovascular AMD was the study eye. The study eye had to have adequate media clarity and pupillary dilation and no other ocular comorbidities that could affect retinal imaging or the course of AMD.

### Assessments

The data collected at baseline included age, gender, ethnicity, smoking status, and visual acuity for both eyes. Race and ethnicity were self-reported by the participants and categorized by researchers as Black, South Asian (which included Bangladeshi, Indian, or Pakistani), other Asian (individuals who were not Bangladeshi, Chinese, Indian, or Pakistani), White, or any individual ethnicity not named in the UK 2011 Census. Smoking status were defined as nonsmoker, previous smoker, and current smoker.

The OCT and OCTA captured at baseline plus or minus 3 months of the first anti-VEGF injection were collected and graded. All eyes were then prospectively imaged, as per study protocol, for their year 1 and 2 visits. Spectralis OCT/Heidelberg retina angiograph with OCT (Heidelberg Engineering) was used in 20 sites and 5 sites that used Topcon OCT-A (Topcon 3D OCT-1000 or Topcon OCT −2000 series version 11.1 or higher). Please see eTable 1 in Supplement 1 for image capture protocols for DLS and neMNV

#### Grading of DLS on OCT and neMNV on OCT-A.

The retinal images linked to the demography and clinical data of each patient from the participating sites were transferred to Moorfields Eye Hospital for grading by trained medical retina fellows who were clinical practitioners in retinal clinics. Please see eTable 1 in Supplement 1 for grading protocols for DLS and neMNV. The DLS were also graded by their height to thick and thin DLS.^22,23^ The fellows were trained by the senior author (S.S.) together on 20 datasets having both case-positive and case-negative cases of DLS and then each of the 4 graders (S.T., S.K, D.K., and A.M.K.) completed independent grading of presence or absence of DLS and neMNV on 20 eyes for both intra- and interobserver grading until a κ agreement of at least 0.7 was achieved with repeated training (eTable 2 in Supplement 1). In addition, all neMNV were graded twice by 2 graders (S.T. and S.S.) due to the challenges of localizing these lesions.^26^

#### Grading of Other AMD Features on OCT

Hyperreflective foci (HRF) were defined as scattered, focal, punctate hyperreflective lesions (equal to or greater than the RPE reflectivity) observed within the neurosensory retina. Presence or absence of ellipsoid layer (EZ)/external limiting membrane (ELM) were graded. Presence of complete atrophy was defined as per Classification of Atrophy Meeting definition.^27,28^

#### Outcomes

The primary outcome was prevalence of neMNV in the study eye. Secondary outcomes included prevalence of DLS and neMNV in DLS. Post hoc analysis of the associations of thick and thin DLS with neMNV were also performed.

#### Statistical Analysis

The analysis sample included only those with both OCT and OCT-A performed within 30 days of the date of anti-VEGF injection. Baseline demographic, clinical, and ocular characteristics were summarized for the overall cohort using mean (SD) or median (IQR) for continuous variables and counts with percentages for categorical variables. The final number of participants analyzed, including their distribution of DLS and neMNV status at baseline was illustrated using a flowchart. The proportion of DLS and neMNV were summarized as point estimates with corresponding 95% CIs estimated using the Wilson score interval with continuity correction. Participant characteristics were further stratified by neMNV status, DLS status and thickness of DLS (DLS height >40 um threshold for thick DLS). Descriptive summaries and statistical comparisons of all study variables were also performed, stratified by neMNV within subgroups of eyes with DLS, as well as within eyes with thick DLS. Participant characteristics of those that were excluded due to OCT and OCT-A not being performed within 30 days of the first fellow eye anti-VEGF injection were summarized to evaluate potential differences in distribution relative to those included in the main analysis.

Group comparisons were performed using independent-samples *t* tests for continuous variables with welches correction for unequal variance and Pearson χ^2^ test for categorical variables, with continuity corrected applied for 2 × 2 tables. When expected cell counts were less than 5, Fisher-exact test was used. For group comparisons with categorical variables, risk difference with 95% CIs were calculated using the Newcombe method based on the Wilson score interval using continuity correction to improve robustness, particularly for proportions near 0 or 1. For continuous variables, mean difference with 95% CIs were reported. Associations between study variables with the presence of DLS and neMNV at baseline were assessed using unadjusted and adjusted logistic regression models. Adjusted models were fitted separately with age (continuous), gender, and ethnicity included as covariates to adjust for potential confounding.

## Results

A total of 862 participants were considered eligible at baseline, of whom 550 (63.8%) had both OCT and OCT-A performed within 30 days of the date of the first anti-VEGF injection to the fellow eye (eFigure 1 in Supplement 1). Participant characteristics of these 550 eyes were comparable with those excluded due to OCT and OCT-A not being performed within the allowed window in all eyes; however, there was a lower proportion of non-White ethnicity in those that were excluded (n = 6; 2.5% vs n = 36; 6.6%; *P* = .02) (eTable 3 in Supplement 1).

### Comparison of Participants by DLS Status and neMNV Status

The mean (SD) age was 78.0 (7.6) years and 315 (57.3%) participants were female. At baseline, 112 eyes (20.4%; 95% CI [Wilson score], 17.1%-24.0%) had DLS, and 47 eyes (8.5%; 95% CI [Wilson score], 6.4%-11.3%) had neMNV. The proportion of DLS was higher in female participants (n = 76 [24.1%]) compared with male participants (n = 36 [15.3%]; *P* = .02) (Table). Among the 47 eyes with neMNV at baseline, 42 (89.4%) were within DLS, 3 (6.4%) in FIPED, and 2 (4.3%) below drusen. When eyes with and without neMNV were compared, mean overall age did not differ between groups. However, the distribution of age categories differed between groups, with a higher proportion of neMNV observed in participants from the younger than 65 years age group (n = 5 [22.7%]) compared with those aged 65 to 80 years (n = 21 [7.0%]) and older than 80 years (n = 21 [9.1%]; *P* = .04).

**Table.** Participant Characteristics Overall and by Double-Layer Signs (DLS) and Nonexudative Macular Neovascularization (neMNV) Status

Variable	DLS status				neMNV status			
	No. (%)^a^			*P* value^b^	No. (%)^a^			*P* value^b^
	Absent (n = 438)	Present (n = 112)	Difference, % (95% CI)		Absent (n = 503)	Present (n = 47)	Difference, % (95% CI)	
Age categories, y								
<65	14 (63.6)	8 (36.4)	1 [Reference]	.08	17 (77.3)	5 (22.7)	1 [Reference]	.04
65-80	245 (82.2)	53 (17.8)	−18.6 (−41.8 to 0.4)		277 (93.0)	21 (7.0)	−15.7 (−38.9 to −1.2)	
>80	179 (77.8)	51 (22.2)	−14.2 (−37.6 to 5.1)		209 (90.9)	21 (9.1)	−13.6 (−36.9 to 1.2)	
Age, y, mean (SD)	77.9 (7.2)	78.6 (9.0)	0.7 (−1.1 to 2.5)	.44	78.1 (7.4)	77.8 (9.8)	−0.3 (−3.2 to 2.7)	.86
Gender								
Female	239 (75.9)	76 (24.1)	1 [Reference]	.02	285 (90.5)	30 (9.5)	1 [Reference]	.43
Male	199 (84.7)	36 (15.3)	−8.8 (−15.5 to −1.8)		218 (92.8)	17 (7.2)	−2.3 (−7.1 to 2.9)	
Self-reported ethnicity^c^								
Non-White^d^	29 (80.6)	7 (19.4)	−1.0 (−12.3 to 16.4)	1.00	33 (91.7)	3 (8.3)	−0.2 (−7 to 15.2)	1.00
White	408 (79.5)	105 (20.5)	1 [Reference]		469 (91.4)	44 (8.6)	1 [Reference]	
Smoker status								
Nonsmoker	249 (79.6)	64 (20.4)	1 [Reference]	.23	284 (90.7)	29 (9.3)	1 [Reference]	.82
Former smoker	155 (82.0)	34 (18.0)	−2.5 (−9.6 to 5.2)		174 (92.1)	15 (7.9)	−1.3 (−6.4 to 4.5)	
Current smoker	34 (70.8)	14 (29.2)	8.7 (−4.1 to 24.4)		45 (93.8)	3 (6.3)	−3 (−9.1 to 9.3)	
BCVA study eye, ETDRS letter score, mean (SD) [Snellen Equivalent at 20 ft]	76.1 (9.8) [20/25 to 20/32]	75.2 (10.0) [20/25 to 20/32]	−0.9 (−3.0 to 1.2)	.39	75.8 (10.0)[20/25 to 20/32]	77.8 (8.1) [20/25 to 20/32]	2.0 (−0.6 to 4.5)	.12
Drusen								
Absent	62 (83.8)	12 (16.2)	1 [Reference]	.43	70 (94.6)	4 (5.4)	1 [Reference]	.42
Present	376 (79.0)	100 (21.0)	4.8 (−6.6 to 13)		433 (91.0)	43 (9.0)	3.6 (−5.3 to 8.4)	
Ellipsoid zone or ELM loss								
Absent	399 (81.6)	90 (18.4)	1 [Reference]	.002	447 (91.4)	42 (8.6)	1 [Reference]	1.00
Presence	39 (63.9)	22 (36.1)	17.7 (5.5 to 31.4)		56 (91.8)	5 (8.2)	−0.4 (−6.3 to 10.5)	
Hyperreflective foci								
Absent	334 (84.6)	61 (15.4)	1 [Reference]	<.001	364 (92.2)	31 (7.8)	1 [Reference]	.44
Presence	104 (67.1)	51 (32.9)	17.5 (9.2 to 26.2)		139 (89.7)	16 (10.3)	2.5 (−2.8 to 9.1)	
Atrophy								
Absent	376 (81.2)	87 (18.8)	1 [Reference]	.07	420 (90.7)	43 (9.3)	1 [Reference]	.23
iRORA	36 (75.0)	12 (25.0)	6.2 (−5.4 to 21.5)		47 (97.9)	1 (2.1)	−7.2 (−10.9 to 3.5)	
cRORA	26 (66.7)	13 (33.3)	14.5 (0.3 to 31.9)		36 (92.3)	3 (7.7)	−1.6 (−8.1 to 12.9)	

Abbreviations: BCVA, best-corrected visual acuity; CONAN, Consensus on Neovascular Age-Related Macular Degeneration Nomenclature; cRORA, complete retinal pigment epithelium and outer retinal atrophy; ELM, external limiting membrane; ETDRS, Early Treatment Diabetic Retinopathy Study; iRORA, incomplete retinal pigment epithelium and outer retinal atrophy; SDD, subretinal drusenoid deposits.

^a^ Row proportions presented for categorical variables.

^b^ Two-sample *t* test with Welch correction for continuous variables; Pearson χ^2^ test for categorical variables, with continuity correction for 2 × 2 tables and risk difference with 95% CIs based on the Newcombe method using Wilson score interval with continuity correction; Fisher exact test for categorical variables where expected cell count less than 5.

^c^ Ethnicity missing in 1 eye.

^d^ Includes participants who self-identified as Black (n = 7), South Asian or any other Asian (n = 24), and mixed or other race (n = 5).

Owing to variability in Heidelberg OCT acquisition protocols, study eyes were classified according to scan density, differentiating those assessed for DLS with 49 or more line scans from those evaluated with less than 49-line scans. Using higher-density OCT protocols (≥49 line scans), DLS was detected in 27 of 129 eyes (20.9%) eyes vs 85 of 421 (20.2%) in eyes imaged with lower-density protocols (<49-line scans) (*P* = .95). In addition, as neMNV prevalence was assessed using either Heidelberg OCT-A or Topcon OCT-A, analyses were stratified by imaging platform, with neMNV detected in 28 of 365 (7.7%) for Heidelberg OCT-A and 19 of 185 (10.3%) for Topcon OCT-A (*P* = .39). Intergrader agreement was high, with κ values ranging between 0.6 and 1.0 (eTable 2 in Supplement 1) while intragrader agreement was done in 2 graders and averaged 0.8. Ambiguous neMNV cases and instances of intergrader disagreement were resolved by arbitration after manual review and consensus, involving 4 cases imaged with the Heidelberg OCT-A system and 3 cases imaged with the Topcon OCT-A system.

The presence of DLS was also higher in those with EZ/ELM loss (n = 22 [36.1%]) than in those without (n = 90 [18.4%]; *P* = .002) (Table). Similarly, eyes with HRF more frequently observed DLS (n = 51; 32.9%) compared with eyes without HRF (n = 61 [15.4%]; *P* < .001).

### Comparison of Characteristics by neMNV Status in Eyes With DLS

**Figure 1.**
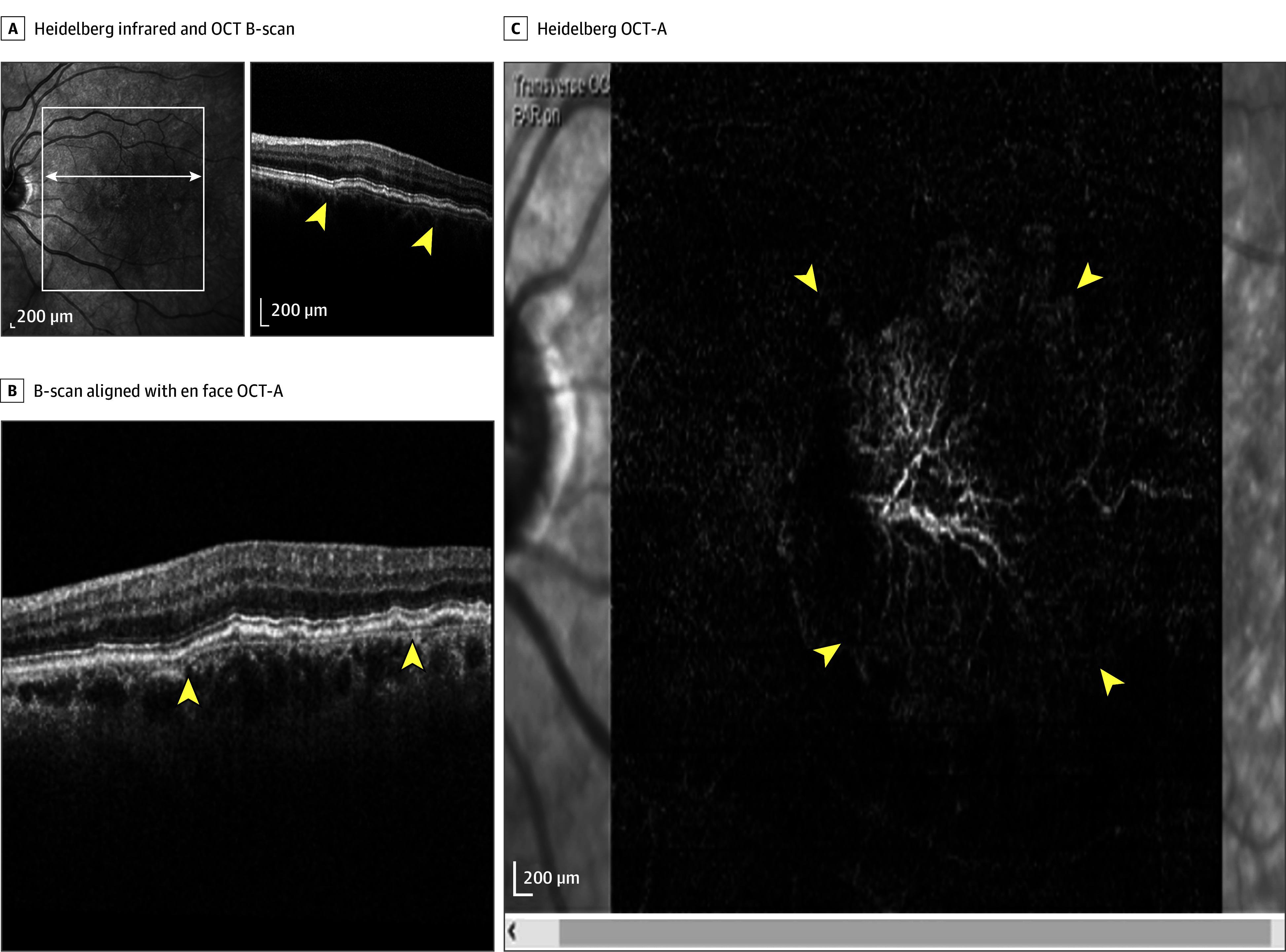
Imaging Showing a Thick Double-Layer Sign That Contains a Nonexudative Neovascular Membrane A, Heidelberg infrared and optical coherence tomography (OCT) brightness scan (B-scan) shows the double-layer sign and the boundary is indicated by yellow arrowheads. B, The B-scan that aligns with the enface OCT angiography (OCT-A) image and the boundary is indicated by yellow arrowheads. C, Heidelberg OCT-A shows the nonexudative neovascular membrane and the boundary of choroidal neovascularization is indicated by yellow arrowheads.

**Figure 2.**
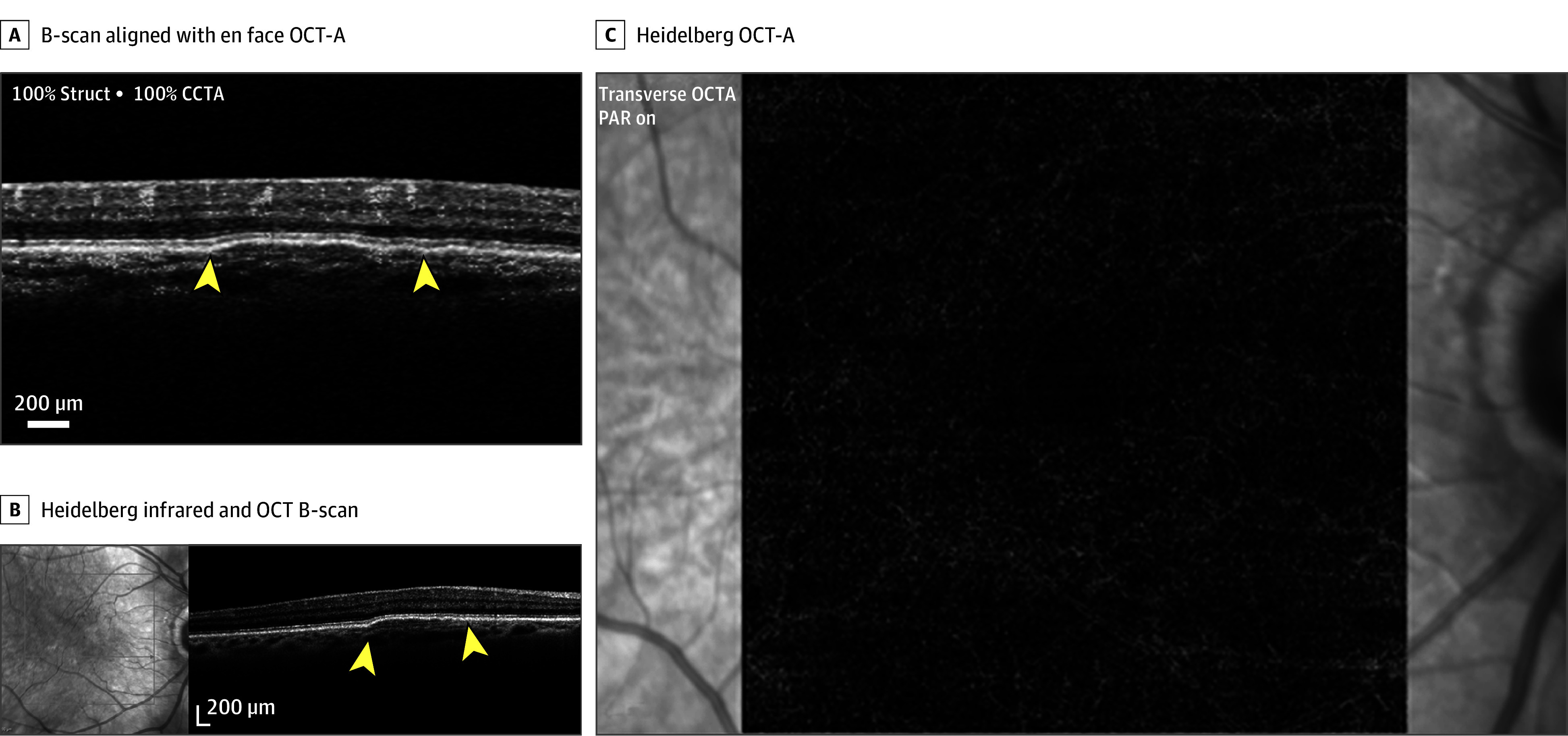
Image Showing a Thick Double-Layer Sign That Does Not Contain a Nonexudative Neovascular Membrane A, The brightness scan (B-scan) that aligns with the enface optical coherence tomography angiography (OCT-A) image and the boundary is indicated by yellow arrowheads. B, Heidelberg OCT-A shows the absence of nonexudative neovascular membrane. C, Heidelberg infrared and OCT B-scan shows the double-layer sign and the boundary is indicated by yellow arrowheads. CCTA indicates coronary computed tomography angiography; PAR, persistent avascular retina.

**Figure 3.**
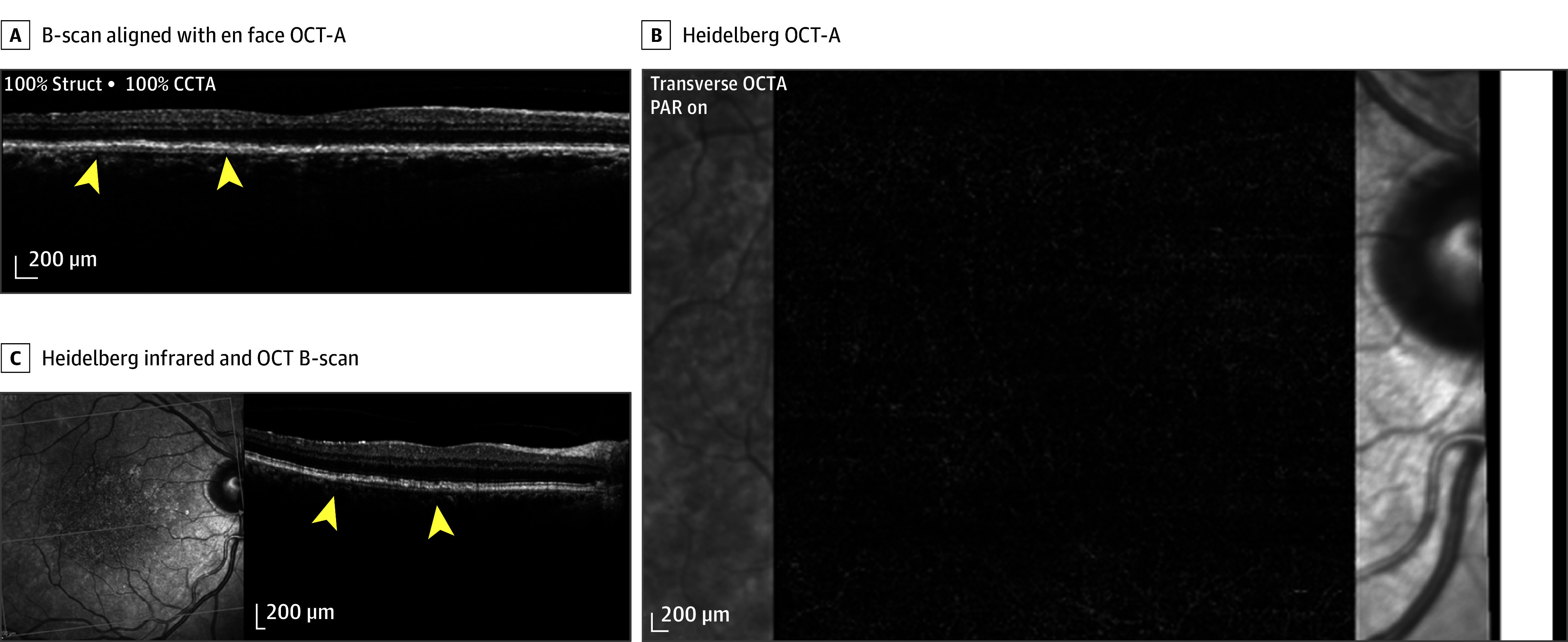
Imaging Showing a Thin Double-Layer Sign That Does Not Contain a Nonexudative Neovascular Membrane A, The brightness scan (B-scan) that aligns with the enface optical coherence tomography angiography (OCT-A) image and the boundary is indicated by yellow arrowheads. B, Heidelberg OCT-A showing the absence of nonexudative neovascular membrane. C, Heidelberg infrared and OCT B-scan showing the double-layer sign and the boundary is shown indicated by yellow arrowheads. CCTA indicates coronary computed tomography angiography; PAR, persistent avascular retina.

**Figure 4.**
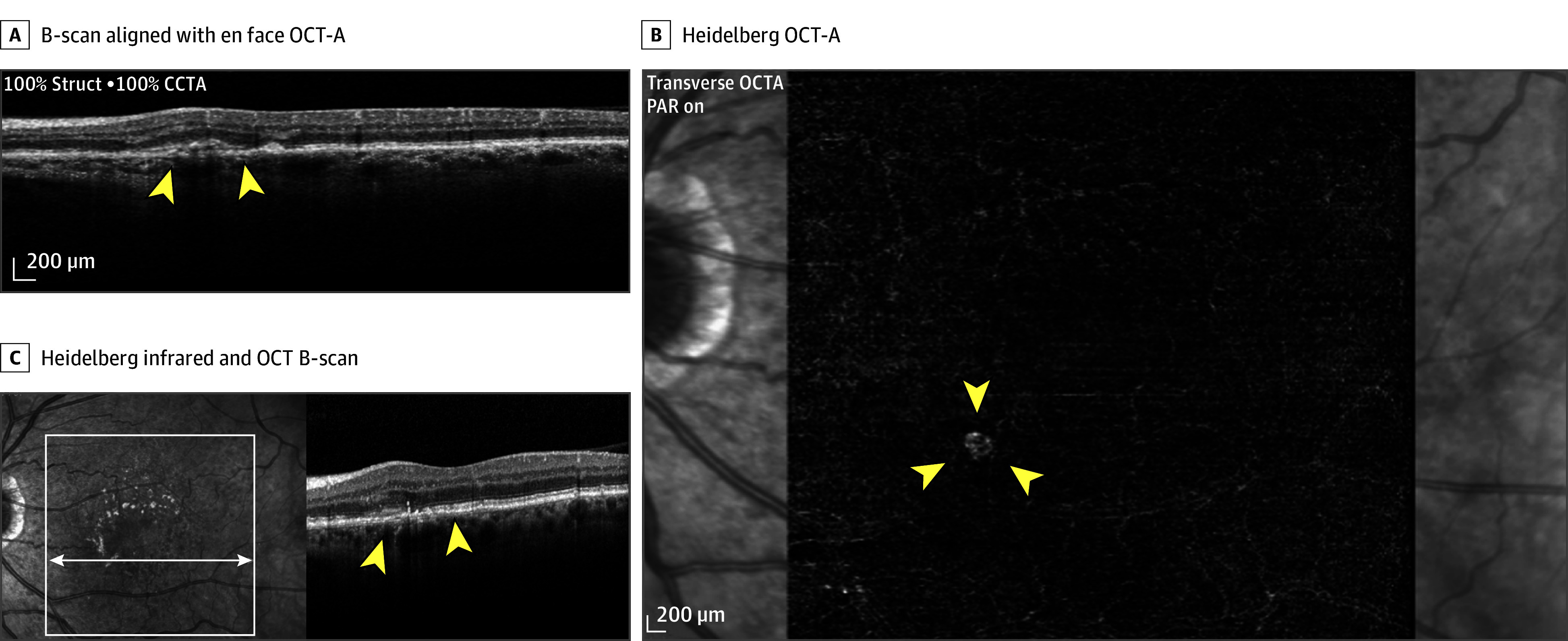
Imaging Showing a Thin Double-Layer Sign That Contains a Nonexudative Neovascular Membrane A, The brightness scan (B-scan) that aligns with the enface optical coherence tomography angiography (OCT-A) image and the boundary is indicated by yellow arrowheads. B, Heidelberg OCT-A showing the nonexudative neovascular membrane and the boundary of choroidal neovascularization is indicated by yellow arrowheads. C, Heidelberg infrared and OCT B-scan showing the double-layer sign and the boundary is indicated by yellow arrowheads. CCTA indicates coronary computed tomography angiography; PAR, persistent avascular retina.

In 47 eyes with neMNV, 42 were located within the DLS, where mean (SD) height was 61.8 (18.7) um compared with 50.9 (19.6) um in the 70 eyes with neMNV absent (*P* *=* .005) (eTable 4 in Supplement 1) (for examples, see Figure 1, Figure 2, Figure 3, and Figure 4). No difference was observed in DLS length and all eyes with neMNV, in this group with DLS, presented with heterogeneous reflectivity of contents (eTable 4 in Supplement 1). Additionally, 96 (85.7%) met the definition of thick DLS based on a height cutoff of more than 30 um while 75 (67.0%) met the definition based on a more than 40 um cutoff. Using the more than 40 um threshold, the prevalence of neMNV was higher among eyes with thick DLS (n = 36 [48.0%]) than among eyes with thin DLS (n = 6 [16.2%]; *P* = .002). In contrast, when applying the more than 30 um cutoff, the difference in neMNV prevalence was less pronounced (40.6% [n = 39] in thick DLS vs 18.8% [n = 3] in thin DLS; *P* = .16).

### Univariable and Adjusted Associations for the Presence of DLS and neMNV Using Logistic Regression Models

EZ/ELM loss (age, gender, and ethnicity adjusted odds ratio [OR], 2.51; 95% CI, 1.40-4.47; *P* = .002) and presence of HRF (adjusted OR, 2.61; 95% CI, 1.68-4.06; *P* < .001) was associated with increased odds of any DLS (eFigure 2 and eTable 5 in Supplement 1). The presence of neMNV was not associated with any OCT factors (eFigure 2 and eTable 6 in Supplement 1).

### Comparison of Participant Characteristics Stratified by Thin or Thick DLS and the Distribution of neMNV Within Thick DLS

Participant characteristics stratified by the presence of thick DLS (defined as 40-100 mm) are presented in eTable 7 in Supplement 1. Participants with thick DLS were markedly younger (mean [SD] age, 77.3 [9.1] years) than those with thin DLS (81.2 [8.2] years; *P* = .02). Thick DLS was more prevalent in eyes with neMNV (n = 36 [85.7%]) compared with those with absent neMNV (n = 39 [55.7%]; *P* = .002). Nearly all eyes with homogenous reflectivity had thin DLS (n = 31 [96.9%]), whereas thin DLS was observed in only 7.5% (n = 6) of eyes with heterogenous reflectivity (*P* < .001).

## Discussion

The key finding of this study is that neMNV was identified on OCT-A in only 8.6% of fellow eyes of patients with unilateral, newly diagnosed exudative AMD. In contrast, DLS were observed in 112 eyes (20.4%), consistent with previously reported baseline DLS prevalence.^19,21^As neMNV was present in only 40% of eyes with DLS, these findings suggest that DLS on structural OCT may not be a good surrogate marker for neMNV.

As previously reported, grading of neMNV on OCT-A is challenging, particularly for small lesions and in the presence of imaging artifacts or coexisting pathologic features, such as drusen and atrophy, often necessitating slab manipulation. To mitigate these limitations and ensure grading accuracy, all eyes with neMNV were independently regraded by 2 graders, with discrepancies resolved by consensus. Additionally, because 2 OCT-A devices were used in this study, neMNV prevalence was evaluated by device and found to be similar across platforms, at approximately 8% to 10%.

In this study, approximately 90% of neMNV were located within thick DLS but about 50% of thick DLS did not harbor neMNV. In addition, we did not find an association of length of DLS of more than 1000 micrometers as a risk factor for neMNV.^18,21^ Moreover, the arbitrary cutoff of height of DLS of less than 100 micrometers excluded 3 eyes where the RPE-Bruch membrane separation slightly exceeded 100 micrometers. We termed this group as having FIPED as seen in central serous chorioretinopathy. Therefore, neither the height or length of DLS can be used to accurately diagnose neMNV.

Heterogeneity of reflectivity of the content of DLS is also considered a marker of neMNV but only 50% of DLS with heterogeneous reflectivity harbored neMNV. These observations suggest that although thick DLS is a known predictor of neMNV and exudative MNV; almost half the eyes with thick DLS may only harbor BLD or have neMNV with slow flow that cannot be detected on OCT-A.

It is interesting to note that neMNV were also detected in eyes with thin DLS (16.2%). Although most thin DLS may indeed only contain BLD and are more prone to atrophy, this study revealed the importance of monitoring these eyes for neMNV, too.^23^ However, it is challenging to grade thin DLS as the medium to low reflectivity of the contents may not always be gradable, consistent with previous report by Sura et al.^23^

Interestingly, both thick DLS and neMNV were more common in the younger age group (<65 years) and the prevalence of neMNV decreased with increasing age. Females were found to have more DLS than males and this may explain why females transition to exudative nAMD in the fellow eye of nAMD earlier than males.^29^ Hyperreflective foci was also associated with presence of DLS corroborating with previous reports that they are predisposing signs for both atrophy and exudative nAMD.^30^ Loss of ellipsoid/ELM layers, an early sign of outer retinal damage, was also associated with DLS but none of these OCT characteristics were associated with neMNV. These observations may demonstrate the protective effect of neMNV against development of atrophy in about 10% of eyes at time of diagnosis of unilateral nAMD.

Several researchers have reported the protective effect of neMNV on the outer retina and RPE by providing metabolic support and oxygenation in eyes with atrophy.^14,16,31^ Therefore, intervention to prevent exudation of neMNV may prolong time to atrophy and protect an eye from both types of late AMD. Based on our study, if patients are recruited for such interventions to delay exudation from neMNV at baseline, defined as time of first injection for unilateral nAMD, only about 14% eyes will have thick DLS and 50% of these will have neMNV. Given the low prevalence of neMNV at baseline, it may be more appropriate to include eyes with thick DLS with neMNV throughout the course of anti-VEGF therapy for the other eye to enrich the population.

### Strengths and Limitations

Our study has several strengths. EYENEON is a prospective multicenter study conducted across 25 National Health Service sites, highlighting the generalizability of the findings. The presence of neMNV is confirmed by OCT-A. The grading of DLS and dimensions were double-graded and all cases were reviewed by the senior author (S.S.).

The primary limitation of this study is its multicenter design, which permitted retrospective image collection at baseline and resulted in OCT scans acquired using local protocols with variable scan densities and use of 2 types of OCT-A devices. To address this heterogeneity and enhance clinical generalizability, we reported the prevalence of DLS and neMNV stratified by OCT scan protocols and OCT-A platforms. Despite these measures, limitations in the reliability and reproducibility of neMNV detection remain, particularly in eyes with drusen or fibrovascular pigment epithelial detachments and when assessing the entire macula and should be considered when interpreting the results. In addition, fluorescein angiography may detect neMNV not identified by OCT-A.

## Conclusions

Although 20% of fellow eyes of unilateral new-onset exudative AMD have DLS, only 8.6% have neMNV, highlighting the need to diagnose neMNV using OCT-A than rely on the characteristics of DLS. These observations may partly explain the interindividual variations in rate of transition to exudative nAMD in the fellow eyes of people with unilateral nAMD on anti-VEGF therapy.

## Notes

Supplement 1. **eFigure 1.** Participant flow **eTable 1.** Grading Protocols **eFigure 2.** Forest plot of adjusted Odds Ratios (aOR) with 95% CI from logistic regression models evaluating factors associated with presence of DLS and neMNV at baseline **eTable 2.** Inter-grader agreement (pairwise Kappa values) for DLS, thick/thin DLS, and neMNV on 20 eyes **eTable 3.** Comparison of participants with missing OCTA and OCT within 30 days of injection vs complete data included in the final analysis **eTable 4.** Characteristics by neMNV status in eyes with DLS **eTable 5.** Univariate and adjusted analysis of presence of DLS using logistic regression models **eTable 6.** Univariate and adjusted analysis of presence of neMNV using logistic regression models **eTable 7.** Comparison of characteristics of eyes with thin vs thick DLS and distribution of neMNV in eyes with thick DLS

Supplement 2. Nonauthor collaborators

Supplement 3. Data sharing statement
