# Supplementary material for: Nonexudative Macular Neovascularization in Age-Related Macular Degeneration
Source: JAMA Ophthalmol. 2026 Apr 9;144(5):405–13. doi: 10.1001/jamaophthalmol.2026.0459 (PMC13067133; doi:10.1001/jamaophthalmol.2026.0459)
Supplement: Supplement 1. — eFigure 1. Participant flow eTable 1. Grading Protocols eFigure 2. Forest plot of adjusted Odds Ratios (aOR) with 95% CI from logistic regression models evaluating factors associated with presence of DLS and neMNV at baseline eTable 2. Inter-grader agreement (pairwise Kappa values) for DLS, thick/thin DLS, and neMNV on 20 eyes eTable 3. Comparison of participants with missing OCTA and OCT within 30 days of injection vs complete data included in the final analysis eTable 4. Characteristics by neMNV status in eyes with DLS eTable 5. Univariate and adjusted analysis of presence of DLS using logistic regression models eTable 6. Univariate and adjusted analysis of presence of neMNV using logistic regression models eTable 7. Comparison of characteristics of eyes with thin vs thick DLS and distribution of neMNV in eyes with thick DLS [file jamaophthalmol-e260459-s001.pdf]

Supplemental Online Content

Thottarath S, Gurudas S, Kubravi S, et al; EYENEON Study Group. Nonexudative macular neovascularization in age-related macular degeneration. *JAMA Ophthalmol*. Published online March 26, 2026.
doi:10.1001/jamaophthalmol.2026.0459

eFigure 1. Participant flow
eTable 1. Grading Protocols
eFigure 2. Forest plot of adjusted Odds Ratios (aOR) with 95% CI from logistic regression models evaluating factors associated with presence of DLS and neMNV at baseline
eTable 2. Inter-grader agreement (pairwise Kappa values) for DLS, thick/thin DLS, and neMNV on 20 eyes
eTable 3. Comparison of participants with missing OCTA and OCT within 30 days of injection vs complete data included in the final analysis
eTable 4. Characteristics by neMNV status in eyes with DLS
eTable 5. Univariate and adjusted analysis of presence of DLS using logistic regression models
eTable 6. Univariate and adjusted analysis of presence of neMNV using logistic regression models
eTable 7. Comparison of characteristics of eyes with thin vs thick DLS and distribution of neMNV in eyes with thick DLS

This supplemental material has been provided by the authors to give readers additional information about their work.

© 2026 Thottarath S et al. *JAMA Ophthalmol*.

eFigure 1. Participant flow

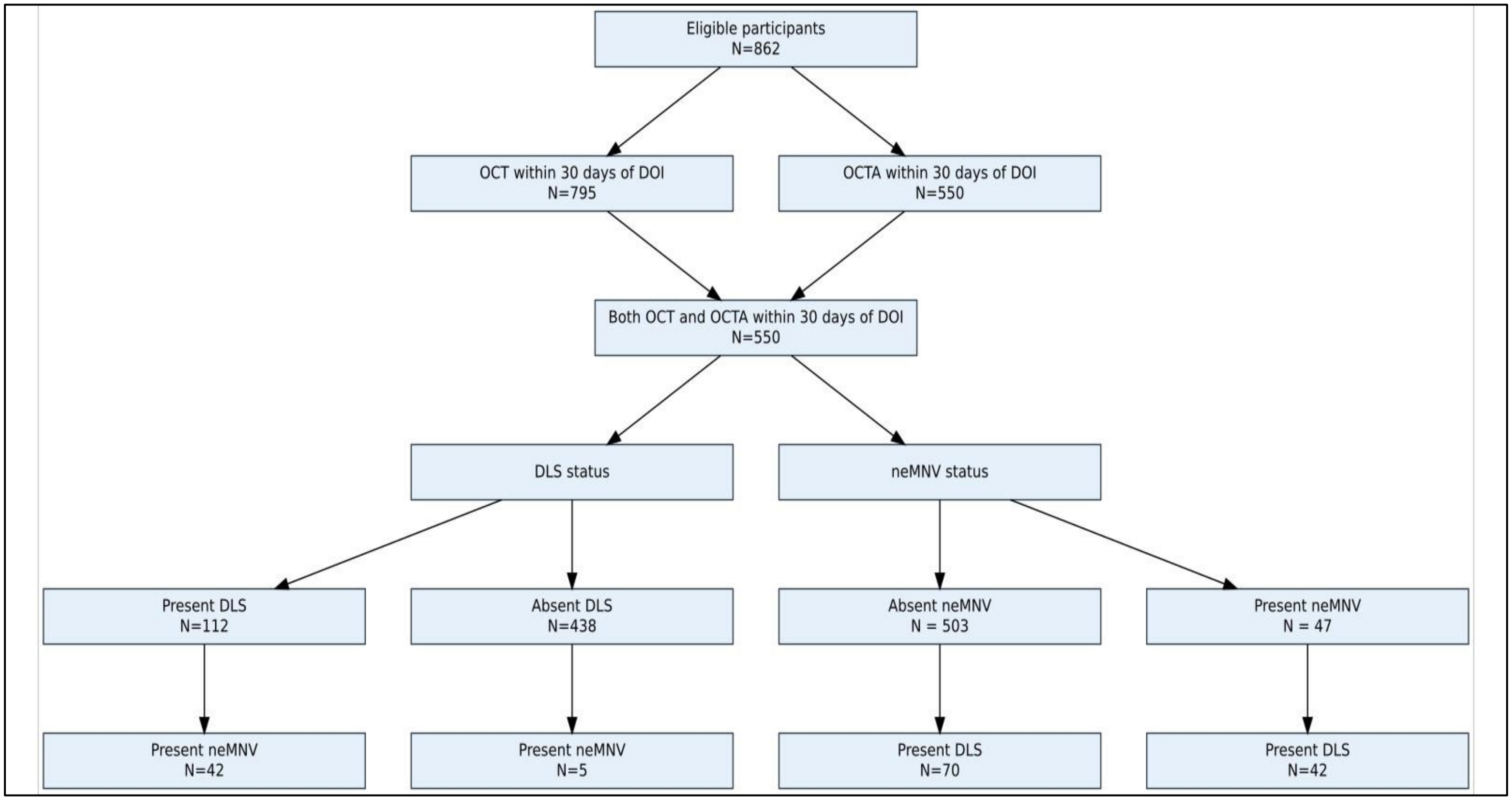

Abbreviations: DLS-Double layer sign; neMNV-non exudative macular neovascularization; OCT-optical coherence tomography; OCTA- optical coherence tomography angiography; DOI-date of injection

© 2026 Thottarath S et al. *JAMA Ophthalmol*.

eTable 1. Grading Protocols

Image capture protocol for Heidelberg OCT and OCTA	The raster scan pattern across the 20-degree field of view varied between 21-49 B-scans. The OCTA module of the standard Heidelberg Eye Explorer (HEYEX) software (v.1.9.14.0) was used to capture a high speed 20° scan (HS20). In addition, a high resolution 10° scan (HR10) was also obtained at some sites. The quality criteria of excellent signal strength (> 30dB).
Image capture protocol for Topcon OCT and OCTA	For the five sites that used Topcon OCTA, (Topcon 3D OCT-1000 or Topcon OCT -2000 series version 11.1 or higher), OCT 3D macula 7x7mm cube scan and OCTA 6x6 mm cube scans were acquired.
Grading of DLS	Any degree of separation between the RPE and Bruch's membrane not due to drusen was graded. The B-scan displaying the most significant RPE separation was chosen to measure the extent. The length of the separation was determined by measuring the horizontal boundaries at the nasal and temporal locations where the RPE and BM could no longer be distinguished. The maximum height of the RPE was also measured on the same B-scan. A separation of less than 100μm in height between the RPE and BM was used to define DLS and any flat irregular separation that exceeded 100μm was defined as FIPED. Graders then examined the heterogeneity of the reflectivity within the DLS but some were too thin to be graded and were categorized as homogenous or too shallow to grade. A cut-off of 30 μm has been reported to distinguish thick from thin DLS. In addition, as a minimum height of 40 microns is required to ensure gradability of hyporeflectivity of core of drusen, we also applied it to DLS. Therefore, both heights were considered to study the prevalence of neMNV within DLS. All DLS were double-graded and disagreement among graders were arbitrated by the senior grader, S.S.
Grading of neMNV on OCTA<br>On Heidelberg OCTA	The presence of neMNV were double-graded based on both flow signals and enface images by two independent graders (S.T. and S.S) because of the challenges of grading these lesions on OCT-A and consensus reached after manual review. The built in 'avascular complex' module on the OCTA tab was used to segment the outer photoreceptor layer and Bruch's membrane. Any abnormal vasculature between these retinal layers could be observed using the "transverse OCT-A" window of the software. If clarification was needed, the OCT-A image was manually reviewed by selecting a "user-defined" slab. In Topcon OCTA, the automated layer segmentation boundaries on the enface projection angiography images were manually changed to better visualize the neovascular complex.
Grading of neMNV on OCTA<br>On Topcon OCTA	In Topcon OCTA, the automated layer segmentation boundaries on the enface projection angiography images were manually changed to better visualize the neovascular complex.

© 2026 Thottarath S et al. *JAMA Ophthalmol*.

eFigure 2. Forest plot of adjusted Odds Ratios (aOR) with 95% CI from logistic regression models evaluating factors associated with presence of DLS and neMNV at baseline[1]

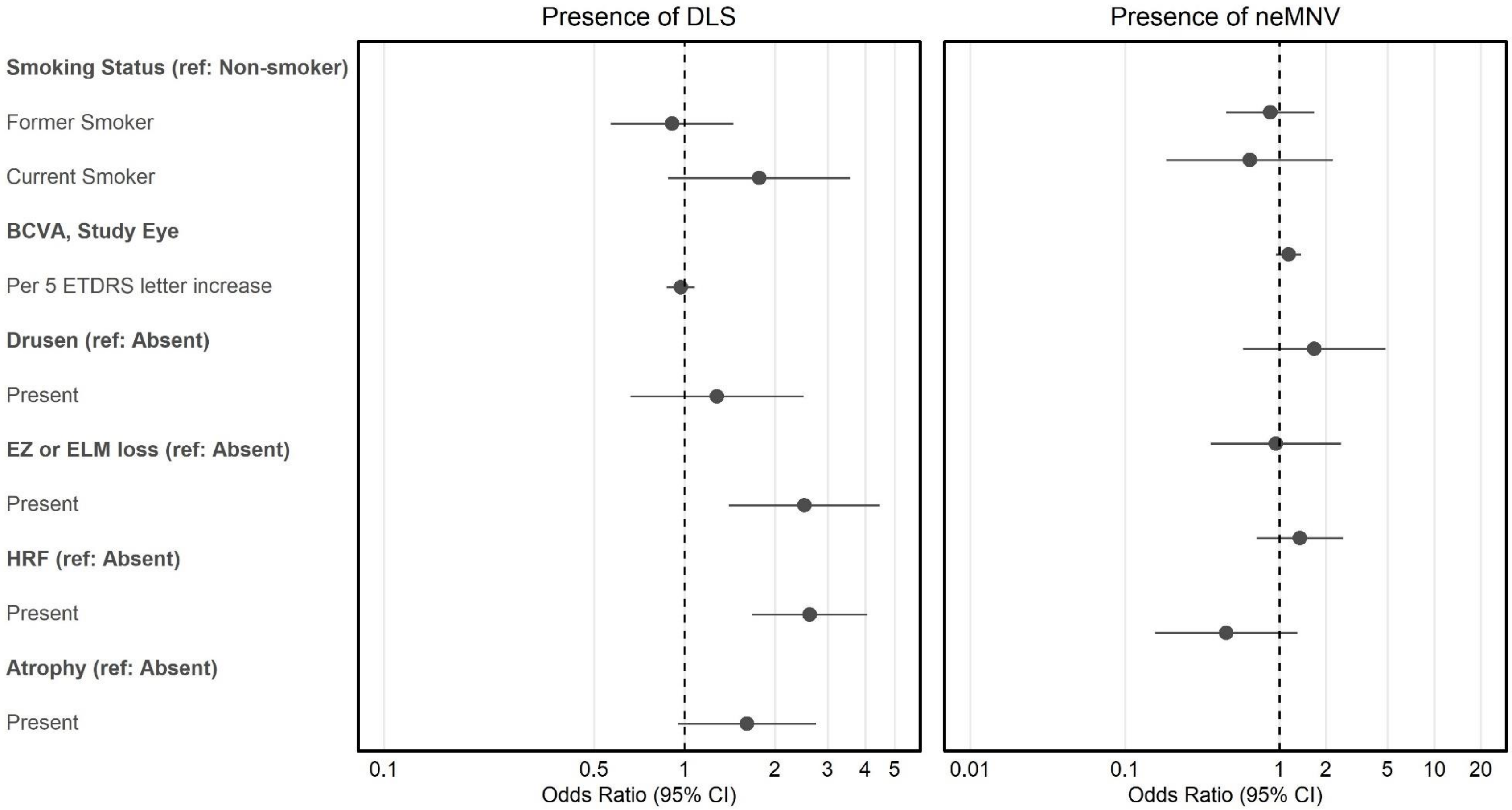

Abbreviations: DLS-Double-layer signs; neMNV-non exudative macular neovascularization; ETDRS-Early Treatment Diabetic Retinopathy Study; BCVA-Best-Corrected Visual Acuity; EZ- Ellipsoid zone; ELM- External Limiting Membrane; HRF- Hyper-reflective foci

[1]Adjusted ORs presented from logistic regression models adjusting for age (continuous), gender and ethnicity. 95% confidence intervals from the forest plot are Wald CIs.

© 2026 Thottarath S et al. *JAMA Ophthalmol*.

eTable 2. Inter-grader agreement (pairwise Kappa values) for DLS, thick/thin DLS, and neMNV on 20 eyes

Kappa values	DLS (Yes/No)	Thick/Thin DLS	neMNV (Yes/No)
**G1/G2**	0.86 [N=20]	0.84 [N=15]	1 [N=20]
**G1/G3**	0.86 [N=20]	0.84 [N=15]	0.9 [N=20]
**G1/G4**	0.74 [N=20]	1 [N=14]	0.8 [N=20]
**G2/G3**	0.73 [N=20]	1 [N=14]	0.9 [N=20]
**G2/G4**	0.88 [N=20]	0.84 [N=14]	0.8 [N=20]
**G3/G4**	0.63 [N=20]	0.83 [N=13]	0.90 [N=20]

Abbreviations: DLS-Double layer sign; neMNV-non exudative macular neovascularization; G1-G4 represents graders.

N denotes the sample size used to calculate the kappa statistics. Thick and thin DLS were graded only in eyes where DLS was assessed as present by that grader; therefore, the total *N* varies.

© 2026 Thottarath S et al. *JAMA Ophthalmol*.

eTable 3. Comparison of participants with missing OCTA and OCT within 30 days of injection vs complete data included in the final analysis

**Variable**	**N**	**Missing**<br>N = 245	**Complete**<br>N = 550
**Age, years, categories**	795		
<65		13 (5.3%)	22 (4.0%)
65-80		124 (50.6%)	298 (54.2%)
>80		108 (44.1%)	230 (41.8%)
**Age, years, Mean (SD)**	795	77.9 (7.2)	78.0 (7.6)
**Gender**	795		
Female		158 (64.5%)	315 (57.3%)
Male		87 (35.5%)	235 (42.7%)
**Ethnicity**	793		
White		238 (97.5%)	513 (93.4%)
non-White		6 (2.5%)	36 (6.6%)
Missing		1	1
**Smoking status**	795		
Non-Smoker		149 (60.8%)	313 (56.9%)
Former Smoker		76 (31.0%)	189 (34.4%)
Current Smoker		20 (8.2%)	48 (8.7%)
**BCVA, SE, ETDRS letter score [Snellen equivalent at 20 feet], Mean (SD)**	795	74.6 (11.6) [20/32 to 20/40]	76.0 (9.8) [20/25 to 20/32]

© 2026 Thottarath S et al. *JAMA Ophthalmol*.

Variable	N	Missing<br>N = 245	Complete<br>N = 550
**BCVA, SE, ETDRS letter score, categories**	795		
>=80		100 (40.8%)	247 (44.9%)
70-79		95 (38.8%)	215 (39.1%)
<=69		50 (20.4%)	88 (16.0%)

Abbreviations: ETDRS-Early Treatment Diabetic Retinopathy Study; BCVA-Best-Corrected Visual Acuity; SD-standard deviation; SE-study eye; SFCT-sub foveal choroidal thickness; CST-central subfield thickness

© 2026 Thottarath S et al. *JAMA Ophthalmol*.

eTable 4. Characteristics by neMNV status in eyes with DLS

Variable, mean(SD) or n(%)	neMNV status: Absent N = 70	neMNV status: Present N = 42	Difference	95% CI	p-value[1]
**DLS height, microns, Mean(SD) [Median, IQR]**	50.9 (19.6) [50.5, IQR 44.0-66.0]	61.8 (18.7) [63.0, IQR 48.0-75.0]	10.9	3.4,18.2	.005
**DLS height, microns, tertiles**					.005
[20,40.3]	31 (83.8%)	6 (16.2%)	Ref	-	
(40.3,65]	18 (50.0%)	18 (50.0%)	33.8%	10.3%,53%	
(65,97]	21 (53.8%)	18 (46.2%)	29.9%	7.2%,48.9%	
**DLS length, microns, mean (SD) [median, IQR]**	1381.0 (856.1) [1179.0, IQR 756.2-1711.0]	1575.1 (1,115.6) [1288.0, IQR 596.2-2114.8]	194.1	-205.3,593.5	.34
**DLS length, microns, categories**					.88
<1000	26 (60.5%)	17 (39.5%)	Ref	-	
>=1000	44 (63.8%)	25 (36.2%)	-3.3%	-22.7%,15.6%	
**DLS length, microns, tertiles**					.63
[278,793]	19 (59.4%)	13 (40.6%)	Ref	-	
(793,1630]	28 (68.3%)	13 (31.7%)	-8.9%	-31.7%,14.4%	
(1630,5210]	23 (59.0%)	16 (41.0%)	0.4%	-23.5%,23.9%	
**DLS categorized by reflectivity of contents**					na
Heterogenous	38 (47.5%)	42 (52.5%)			

© 2026 Thottarath S et al. *JAMA Ophthalmol*.

Variable, mean(SD) or n(%)	neMNV status		Difference	95% CI	p-value[1]
	**Absent** N = 70	**Present** N = 42			
Homogenous	32 (100.0%)	0 (0.0%)			
**DLS categorized by height**					.16
Thick DLS (>30 microns height)	57 (59.4%)	39 (40.6%)	Ref	-	
Thin DLS (<=30 microns height)	13 (81.3%)	3 (18.8%)	-21.9%	-39.2%,7.4%	
**DLS categorized by height**					.002
Thick DLS (>40 microns height)	39 (52.0%)	36 (48.0%)	Ref	-	
Thin DLS (<=40 microns height)	31 (83.8%)	6 (16.2%)	-31.8%	-46.9%,-11.7%	

Abbreviations: DLS-Double layer sign; neMNV-non exudative macular neovascularization; SD-standard deviation; IQR-Interquartile Range.[1]Two sample t-test with welches correction for continuous variables; Pearson's Chi-squared test for categorical variables, with continuity correction for 2x2 tables and risk difference with 95% CIs based on Newcombe's method using Wilson score interval with continuity correction; Fisher's exact test for categorical variables where expected cell count<5.

© 2026 Thottarath S et al. *JAMA Ophthalmol*.

eTable 5. Univariate and adjusted analysis of presence of DLS using logistic regression models

Characteristic	N	Event N	Univariate OR (95% CI)	p-value	Adjusted OR (95% CI)[1]	p-value
**Age, categories, years**	550	112				
<65			—		—	
65-80			0.38 (0.15 - 0.95)	.04	0.39 (0.15 - 0.98)	.046
>80			0.50 (0.20 - 1.25)	.14	0.48 (0.19 - 1.22)	.12
**Age, per 1 year increase**	550	112	1.01 (0.99 - 1.04)	.37	1.01 (0.98 - 1.04)	.52
**Gender**	550	112				
Female			—		—	
Male			0.57 (0.37 - 0.88)	.01	0.57 (0.37 - 0.89)	.01
**Ethnicity**	549	112				
White			—		—	
non-White			0.94 (0.40 - 2.20)	.88	1.00 (0.42 - 2.36)	>.99
**Smoker**	550	112				
Non-Smoker			—		—	
Former Smoker			0.85 (0.54 - 1.35)	.50	0.91 (0.57 - 1.45)	.69
Current Smoker			1.60 (0.81 - 3.16)	.17	1.77 (0.88 - 3.57)	.11
**BCVA, study eye, per 5 ETDRS letters increase**	550	112	0.96 (0.86 - 1.06)	.38	0.97 (0.87 - 1.08)	.63
**Drusen**	550	112				
Absent			—		—	

© 2026 Thottarath S et al. *JAMA Ophthalmol*.

Characteristic	N	Event N	Univariate OR (95% CI)	p-value	Adjusted OR (95% CI)[1]	p-value
Present			1.37 (0.71 - 2.65)	.34	1.28 (0.66 - 2.49)	.46
**EZ or ELM loss**	550	112				
Absent			—		—	
Present			2.50 (1.41 - 4.42)	.002	2.51 (1.40 - 4.47)	.002
**HRF**	550	112				
Absent			—		—	
Present			2.69 (1.74 - 4.14)	<.001	2.61 (1.68 - 4.06)	<.001
**Atrophy**	550	112				
Absent			—		—	
Present			1.74 (1.04 - 2.93)	.04	1.61 (0.95 - 2.74)	.08

Abbreviations: DLS-Double layer sign; BCVA-Best-Corrected Visual Acuity; OR-odds ratio; CI-confidence interval; SDD-subretinal drusenoid deposits; SFCT-sub foveal choroidal thickness; CST-central subfield thickness; EZ-ellipsoid zone; ELM- External Limiting Membrane; HRF-hyperreflective foci; PCV-Polypoidal choroidal vasculopathy.[1] One participant not included in adjusted analysis due to ethnicity being missing (N=549, events=112). Adjusted analysis accounts for age (continuous), gender, and ethnicity. OR with 95% Wald CI and Wald p-values presented.

© 2026 Thottarath S et al. *JAMA Ophthalmol*.

eTable 6. Univariate and adjusted analysis of presence of neMNV using logistic regression models

Characteristic	N	Event N	Unadjusted OR (95% CI)	p-value	Adjusted OR (95% CI)[1]	p-value
**Age, categories, years**	550	47				
<65			—		—	
65-80			0.26 (0.09 - 0.77)	.02	0.26 (0.09 - 0.78)	.02
>80			0.34 (0.11 - 1.02)	.054	0.33 (0.11 - 1.01)	.051
**Age, per 1 year increase**	550	47	1.00 (0.96 - 1.04)	.82	0.99 (0.96 - 1.03)	.75
**Gender**	550	47				
Female			—		—	
Male			0.74 (0.40 - 1.38)	.34	0.73 (0.39 - 1.37)	.33
**Ethnicity**	549	47				
White			—		—	
non-White			0.97 (0.29 - 3.29)	.96	0.98 (0.29 - 3.34)	.97
**Smoker**	550	47				
Non-Smoker			—		—	
Former Smoker			0.84 (0.44 - 1.62)	.61	0.87 (0.45 - 1.68)	.68
Current Smoker			0.65 (0.19 - 2.23)	.50	0.64 (0.19 - 2.22)	.48
**BCVA, study eye, per 5 ETDRS letters increase**	550	47	1.13 (0.94 - 1.35)	.19	1.14 (0.95 - 1.38)	.16
**Drusen**	550	47				
Absent			—		—	

© 2026 Thottarath S et al. *JAMA Ophthalmol*.

Characteristic	N	Event N	Unadjusted OR (95% CI)	p-value	Adjusted OR (95% CI)[1]	p-value
Present			1.74 (0.61 - 4.99)	.30	1.68 (0.58 - 4.85)	.34
**EZ or ELM loss**	550	47				
Absent			—		—	
Present			0.95 (0.36 - 2.50)	.92	0.95 (0.36 - 2.51)	.91
**HRF**	550	47				
Absent			—		—	
Present			1.35 (0.72 - 2.55)	.35	1.35 (0.71 - 2.58)	.36
**Atrophy**	550	47				
Absent			—		—	
Present			0.47 (0.16 - 1.35)	.16	0.45 (0.16 - 1.31)	.14

Abbreviations: neMNV-non exudative macular neovascularization; BCVA-Best-Corrected Visual Acuity; OR-odds ratio; CI-confidence interval; SDD-subretinal drusenoid deposits; SFCT-sub foveal choroidal thickness; CST-central subfield thickness; EZ-ellipsoid zone; ELM- External Limiting Membrane; HRF-hyperreflective foci; PCV-Polypoidal choroidal vasculopathy.[1]One participant not included in adjusted analysis due to ethnicity being missing (N=549, events=112). Adjusted analysis accounts for age (continuous), gender, and ethnicity. OR with 95% Wald CI and Wald p-values presented.

© 2026 Thottarath S et al. *JAMA Ophthalmol*.

eTable 7. Comparison of characteristics of eyes with thin vs thick DLS and distribution of neMNV in eyes with thick DLS

Variable	Thick or thin DLS categorized by DLS height					neMNV status in eyes with thick DLS				
	**Thin DLS (≤40 microns height)** N = 37	**Thick DLS (>40 microns height)** N = 75	**Difference**	**95% CI**	**p-value** [1]	**Absent** N = 39	**Present** N = 36	**Difference**	**95% CI**	**p-value** [1]
**Age, categories, years**					.43					.34
<65	1 (12.5%)	7 (87.5%)	Ref	-		2 (28.6%)	5 (71.4%)	Ref	-	
65-80	17 (32.1%)	36 (67.9%)	-19.6%	-38.2%,22.9%		21 (58.3%)	15 (41.7%)	-29.8%	-58%,15%	
>80	19 (37.3%)	32 (62.7%)	-24.8%	-43.6%,18%		16 (50.0%)	16 (50.0%)	-21.4%	-50.9%,23.4%	
**Age, years**	81.2 (8.2)	77.3 (9.1)	-3.9	-7.3, -0.5	.03	77.7 (8.2)	76.9 (10.1)	-0.7	-5.0,3.5	.74
**Gender**					.79					.46
Female	24 (31.6%)	52 (68.4%)	Ref	-		29 (55.8%)	23 (44.2%)	Ref	-	
Male	13 (36.1%)	23 (63.9%)	-4.5%	-24.8%,14.4%		10 (43.5%)	13 (56.5%)	12.3%	-13.7%,36.1%	
**Ethnicity**					.68					.35
White	34 (32.4%)	71 (67.6%)	Ref	-		38 (53.5%)	33 (46.5%)	Ref	-	
non-White	3 (42.9%)	4 (57.1%)	-10.5%	-48.4%,22.1%		1 (25.0%)	3 (75.0%)	28.5%	-25.9%,55%	
**Smoker**					.48					.17
Non-Smoker	19 (29.7%)	45 (70.3%)	Ref			22 (48.9%)	23 (51.1%)	Ref	-	
Former Smoker	14 (41.2%)	20 (58.8%)	-11.5%	-32.3%,9.1%		9 (45.0%)	11 (55.0%)	3.9%	-23.5%,29.9%	

© 2026 Thottarath S et al. *JAMA Ophthalmol*.

Current Smoker	4 (28.6%)	10 (71.4%)	1.1%	-30.1%,24.1%		8 (80.0%)	2 (20.0%)	-31.1%	-53.3%,7.7%	
**BCVA study eye, ETDRS letter score [Snellen equivalent at 20 feet], mean (SD)**	75.0 (9.4) [20/32]	75.3 (10.3) [20/25 to 20/32]	0.3	-3.5,4.2	.86	73.9 (11.5) [20/32 to 20/40]	76.9 (8.9) [20/25 to 20/32]	3.0	-1.7,7.7	.21
**Drusen**					.06					>.99
Absent	7 (58.3%)	5 (41.7%)	Ref	-		3 (60.0%)	2 (40.0%)	Ref	-	
Present	30 (30.0%)	70 (70.0%)	28.3%	-3.1%,54.9%		36 (51.4%)	34 (48.6%)	8.6%	-36%,43.5%	
**EZ or ELM loss**					.26					.43
Absent	27 (30.0%)	63 (70.0%)	Ref	-		31 (49.2%)	32 (50.8%)	Ref	-	
Present	10 (45.5%)	12 (54.5%)	-15.5%	-39.1%,7.6%		8 (66.7%)	4 (33.3%)	-17.5%	-42.9%,16.3%	
**Reflectivity**					<.001					>.99
Heterogenous	6 (7.5%)	74 (92.5%)	Ref	-		38 (51.4%)	36 (48.6%)	Ref	-	
Homogenous	31 (96.9%)	1 (3.1%)	-89.4%	-94.7%,-72.1%		1 (100.0%)	0 (0.0%)	-48.6%	-60.5%,46.6%	
**HRF**					.06					.84
Absent	15 (24.6%)	46 (75.4%)	Ref	-		23 (50.0%)	23 (50.0%)	Ref	-	
Present	22 (43.1%)	29 (56.9%)	-18.5%	-36%,0.2%		16 (55.2%)	13 (44.8%)	-5.2%	-28.4%,19.1%	
**neMNV**					.002	na				
Absent	31 (44.3%)	39 (55.7%)	Ref	-						
Present	6 (14.3%)	36 (85.7%)	30%	11%,44.9%						
**Atrophy**					.12					.09
Absent	25 (28.7%)	62 (71.3%)	Ref			29 (46.8%)	33 (53.2%)	Ref		
Present	12 (48.0%)	13 (52.0%)	-19.3%	-41.4%,3.2%		10 (76.9%)	3 (23.1%)	-30.1%	-51.2%,3.4%	

© 2026 Thottarath S et al. *JAMA Ophthalmol*.

**DLS height, microns, mean (SD) [Median, IQR]**	32.6 (5.0) [33.0, 30.0-37.0]	66.0 (14.5) [66.0,53.0-75.5]	33.4	29.7,37.1	<.001	65.5 (13.7) [66.0, 54.5-74.0]	66.6 (15.5) [65.0, 52.0-76.3]	1.1	-5.7,7.8	.75
**DLS length, microns, mean (SD) [Median, IQR]**	1,667.9 (1,083.5) [1537.0, 848.0-2120.0]	1,348.1 (883.7) [1188.0, 656.0-1702.5]	-319.7	-730.3,90.9	.12	1,247.6 (760.4) [1070, 676-1610]	1,457.0 (1,000.0) [1257.0, 632.8-1861.0]	209.4	-202.8,621.6	.31
**DLS length, tertiles, microns**					.21					.76
[278,793]	8 (25.0%)	24 (75.0%)	Ref	-		13 (54.2%)	11 (45.8%)	Ref	-	
(793,1630]	12 (29.3%)	29 (70.7%)	-4.3%	-25.2%,18.3%		16 (55.2%)	13 (44.8%)	-1%	-28.5%,26.5%	
(1630,5210]	17 (43.6%)	22 (56.4%)	-18.6%	-39.6%,5.7%		10 (45.5%)	12 (54.5%)	8.7%	-21.6%,37%	
**DLS length, microns, categories**					.48					>.99
<1000	12 (27.9%)	31 (72.1%)	Ref	-		16 (51.6%)	15 (48.4%)	Ref	-	
>=1000	25 (36.2%)	44 (63.8%)	-8.3%	-25.7%,11.1%		23 (52.3%)	21 (47.7%)	-0.7%	-24.3%,22.9%	

Abbreviations: DLS-Double-layer signs; neMNV-non exudative macular neovascularization; ETDRS-Early Treatment Diabetic Retinopathy Study; BCVA-Best-Corrected Visual Acuity; SDD-subretinal drusenoid deposits; SD-standard deviation;; SFCT-sub foveal choroidal thickness; CST-central subfield thickness; EZ-ellipsoid zone; ELM- External Limiting Membrane; HRF-hyperreflective foci; IQR-Interquartile Range; PCV-Polypoidal choroidal vasculopathy.[1]Two sample t-test for continuous variables with welches correction; Pearson's Chi-squared test for categorical variables, with continuity correction for 2x2 tables and risk difference with 95% CIs based on Newcombe's method using Wilson score interval with continuity correction; Fisher's exact test for categorical variables where expected cell count<5.

© 2026 Thottarath S et al. *JAMA Ophthalmol*.
