# Supplementary material for: Nonexudative Macular Neovascularization in Age-Related Macular Degeneration
Source: JAMA Ophthalmol. 2026 Apr 9;144(5):405–13. doi: 10.1001/jamaophthalmol.2026.0459 (PMC13067133; doi:10.1001/jamaophthalmol.2026.0459)
Supplement: Supplement 2. — Nonauthor collaborators [file jamaophthalmol-e260459-s002.pdf]

Supplemental Online Content: Nonauthor Collaborators
*First name, last name, and suffix (if applicable) are required and will appear in PubMed.

*Group Name(s): Eye Neon Study group							
***First Name and Middle Initial(s)**	***Last Name**	***Suffix (eg, Jr, III)**	Academic Degrees	Institution	Location (city, state/province, country)	Role or Contribution, eg, chair, principal investigator	Group (if more than 1 Group listed in the byline) and/or Subgroup (eg, Steering Committee)
Benjamin	Burton		FRCOphth	James Paget University Hospital NHS Foundation Trust	Great Yarmouth, UK	Principal investigator	
Geeta	Menon		FRCOphth	Frimley Health NHS Foundation Trust	Surrey,UK	Principal investigator	
Manju	Chandran		FRCOphth	Frimley Health NHS Foundation Trust	Surrey,UK	Sub investigator	
Ian	Pearce		FRCOphth	Liverpool University Hospital NHS Foundation Trust	Liverpool,UK	Principal investigator	
Savita	Madhusudhan		FRCOphth	Liverpool University Hospital NHS Foundation Trust	Liverpool,UK	Sub investigator	
Anna	Grabowska		FRCOphth	Kings College Hospital NHS Foundation Trust	London,UK	Principal investigator	
Faruque	Ghanchi		FRCOphth	Bradford Teaching Hospital NHS Foundation Trust	Bradford,UK	Principal investigator	
James	Talks		FRCOphth	The Newcastle Upon Tyne Hospital NHS Foundation Trust	Newcastle Upon Tyne,UK	Principal investigator	
Richard	Gale		FRCOphth	York Teaching Hospital NHS Foundation Trust	York, UK	Principal investigator	
Martin	McKibbin		FRCOphth	Leeds Teaching Hospitals NHS Trust	Leeds, UK	Principal investigator	
Ajay	Kotagiri		FRCOphth	South Tyneside And Sunderland NHS Foundation Trust	Sunderland, UK	Principal investigator	
Niro	Narendran		FRCOphth	The Royal Wolverhampton NHS Foundation Trust	Wolverhampton, UK	Principal investigator	
Afsar	Jafree		FRCOphth	East Kent Hospital NHS Foundation Trust	Kent, UK	Principal investigator	
Saad	Younis		FRCOphth	Imperial College NHS Foundation Trust	London, UK	Principal investigator	
Claire	Bailey		FRCOphth	University Hospital Bristol and Weston NHS Foundation Trust	Bristol, UK	Principal investigator	

Supplemental Online Content: Nonauthor Collaborators
*First name, last name, and suffix (if applicable) are required and will appear in PubMed.

***First Name and Middle Initial(s)**	***Last Name**	***Suffix (eg, Jr, III)**	Academic Degrees	Institution	Location (city, state/province, country)	Role or Contribution, eg, chair, principal investigator	Group (if more than 1 Group listed in the byline) and/or Subgroup (eg, Steering Committee)
Priya	Prakash		FRCOphth	The Princess Alexandra Hospital NHS Trust	Harlow, UK	Principal investigator	
Christiana	Dinah		FRCOphth	London North West University Healthcare NHS Trust	Harrow, UK	Principal investigator	
Louise	Downey		FRCOphth	Hull University Teaching Hospital NHS Foundation Trust	Hull, UK	Principal investigator	
Andrew	Lottery		FRCOphth	University Hospital Southampton NHS Foundation Trust	Southampton, UK	Principal investigator	
Paritosh	Shah		FRCOphth	Yeovill District Hospital NHS Foundation Trust	Yeovill, UK	Principal investigator	
Romi	Chhabra		FRCOphth	Manchester University NHS Foundation Trust	Manchester, UK	Principal investigator	
Narendra	Dhingra		FRCOphth	Mid Yorkshire Hospitals NHS Foundation Trust	West Yorkshire, UK	Principal investigator	
Indra	Dias		FRCOphth	Calderdale And Huddersfield NHS Foundation Trust	Huddersfield, UK	Principal investigator	
Mary	Freeman		FRCOphth	Sheffield Teaching Hospital NHS Foundation Trust	Sheffield, UK	Principal investigator	
Daren	Hanumunthadu		FRCOphth	Royal free London NHS Foundation Trust	London, UK	Principal investigator	
