# Supplementary material for: Nonexudative Macular Neovascularization in Age-Related Macular Degeneration
Source: JAMA Ophthalmol. 2026 Apr 9;144(5):405–13. doi: 10.1001/jamaophthalmol.2026.0459 (PMC13067133; doi:10.1001/jamaophthalmol.2026.0459)
Supplement: Supplement 3. — Data sharing statement [file jamaophthalmol-e260459-s003.pdf]

Data Sharing Statement

Thottarath. Nonexudative Macular Neovascularization in Age-Related Macular Degeneration. *JAMA Ophthalmol*. Published April 09, 2026. doi:10.1001/jamaophthalmol.2026.0459

Data
Data available: No

Additional Information
Explanation for why data not available: The data that support the findings of this study are not openly available due to reasons of sensitivity and are available from the corresponding author upon reasonable request
